# Functional connexin35 increased in the myopic chicken retina

**DOI:** 10.1017/S0952523821000079

**Published:** 2021-05-14

**Authors:** Seema Banerjee, Qing Wang, George Tang, ChungHim So, Sze Wan Shan, King Kit Li, Chi-Wai Do, Feng Pan

**Affiliations:** 1 School of Optometry, The Hong Kong Polytechnic University, Kowloon, Hong Kong; 2 School of Clinical Medicine, University of Cambridge, Addenbrooke’s Hospital, Cambridge, United Kingdom; 3 Centre for Eye and Vision Research (CEVR), 17W Hong Kong Science Park, Hong Kong

**Keywords:** myopia, gap junction, retina, amacrine cell

## Abstract

Our previous research showed that increased phosphorylation of connexin (Cx)36 indicated extended  coupling of AII amacrine cells (ACs) in the rod-dominant mouse myopic retina. This research will determine whether phosphorylation at serine 276 of Cx35-containing gap junctions increased in the myopic chicken, whose retina is cone-dominant. Refractive errors and ocular biometric dimensions of 7-days-old chickens were determined following 12 h and 7 days induction of myopia by a −10D lens. The expression pattern and size of Cx35-positive plaques were examined in the early (12 h) and compensated stages (7 days) of lens-induced myopia (LIM). At the same time, phosphorylation at serine 276 (functional assay) of Cx35 in strata 5 (S5) of the inner plexiform layer was investigated. The axial length of the 7 days LIM eyes was significantly longer than that of non-LIM controls (*P* < 0.05). Anti-phospho-Ser276 (Ser276-P)-labeled plaques were significantly increased in LIM retinas at both 12 h and 7 days. The density of Ser276-P of Cx35 was observed to increase after 12 h LIM. In the meanwhile, the areas of existing Cx35 plaques did not change. As there was more phosphorylation of connexin35 at Ser276 at both the early and late stages (12 h) and 7 days of LIM chicken retinal activity, the coupling with ACs could be increased in myopia development of the cone-dominated chicken retina.

## Introduction

Myopia or nearsightedness is the most common refractive error and usually results from excess axial elongation. Its prevalence has been increasing rapidly, with over 80% of adolescents in Hong Kong and 40% of individuals older than 12 years in the United States being myopic (Vitale et al., 2009; Morgan et al., 2012; Yam et al., 2020). Myopia is a risk factor for serious eye diseases, including cataract, glaucoma, retinal detachment, and macular degeneration. Despite the high prevalence and risk of major ocular complications, which make it important to understand what causes the disorder, the etiology of myopia remains poorly understood.

Gap junctions play important functional roles in coding fundamental visual information in the retina. The high plasticity of gap junctions enables the retina to adapt to complicated visual inputs (O’Brien, 2014; O’Brien & Bloomfield, 2018). Connexin (Cx)36 (generally Cx36 in mammals, and its shorter orthologue Cx35 in nonmammals) is present in the coupling between photoreceptors (O’Brien et al., 2012), AII/AII amacrine cells (ACs) (Feigenspan et al., 2001) and is believed to be required for gap junctional coupling of most retina ganglion cell (RGC) subtypes in the mammalian retina (Pan et al., 2010). Gap junctional coupling of AII ACs is modulated through phosphorylation at serine110 and 276/293 (Ser 276 in teleost fish and chicken retina, Ser 293 in mammals) (Kothmann et al., 2009; Kothmann et al., 2012; Meyer et al., 2014). Reports also indicate that the retina plays a major role in refractive development (Wallman & Winawer, 2004; Maiello et al., 2017). Defocusing of images induces changes in firing patterns of RGCs in the mouse retina (Pan, 2019; Banerjee et al., 2020). A possible strategy in reaction to the myopic mouse retina’s noisy signaling status is to increase functional gap junction coupling of AII ACs. It was reported that the phosphorylation state of Ser293, indicative of the function of coupling through Cx36 gap junctions affected by the dopamine pathway, increased in the myopic mouse retina (Banerjee et al., 2020) during refractive development and may be involved in the development of human myopia. Retinal signaling needs to be delivered to the sclera, so phosphorylation of Cx35/36 in the inner plexiform layer (IPL) could be a potential candidate for mediating the retina-to-sclera signaling pathway.

Eye growth in chick (*Gallus gallus*) provides a reliable and rapid model to study mammalian eye growth and human refractive development. The chick model has been used for over 40 years (Wallman et al., 1978; Wisely et al., 2017) and is still the major model for refractive development and myopia study (Schaeffel & Feldkaemper, 2015). Chick eyes are similar to humans, having small lenses and large vitreous chambers (Troilo et al., 2019). Chicks have several advantages as a model because they are readily available, easy to maintain, and rapidly develop myopia (Wisely et al., 2017). In addition, lens-induced myopia (LIM) method can induce precise and high myopia in newborn chicks. The results from up to 1-year-old chicken model used in myopia research correspond developmentally to human adolescence (Papastergiou et al., 1998). However, the retinal circuit is far less known in chicken than that of the mouse. It is arguable whether the AII ACs also existed in chicken (Seifert et al., 2020). Some antibodies might not work in the chicken. All these challenges defer the research in the chicken model.

Specific antibodies labeling the phosphorylated form of Cx35/36 were used to determine the expression patterns of Cx35/36-positive plaques (structural assay) and the state of Cx35/36 phosphorylation (functional assay). The phosphorylation state of Ser276/293 indicates the degree of coupling through Cx35/36 gap junctions in the myopic retina. Cx35/36 phosphorylation leads to an increasing gap junction coupling between AII ACs in the myopic mouse retina (Banerjee et al., 2020). As the cone-dominant and multiplicity of outputs to second-order neurons in the chicken retina are similar to those in humans, findings may also be relevant to understanding myopia in children. The present study showed that phosphorylation of Cx35 gap junctions increased in chicken LIM retina. At the same time, the expression of Cx35 remained unchanged. These results support our hypothesis that Cx35/36 function regulation is critical to understand the visual signaling processes in both normal and myopic retinas.

## Methods

### Ethical approval

All animal procedures were approved by the Animal Subjects Ethics Sub-Committee (ASESC) of the Hong Kong Polytechnic University and complied with the Guide for the Care and Use of Laboratory Animals published by the National Institutes of Health.

### Animals

White Leghorn chickens (*Gallus gallus domesticus*) were hatched from specific pathogen-free eggs (China) (*n* = 28).

Myopia (Zhang et al., 2011; Chun et al., 2015; Zhou et al., 2018) was induced by a negative lens in chicks that were reared in a 12 h light/dark cycle (700 Lux light from 8 AM to 8 PM). Lenses were attached to 25 chicks with −10D on the left eye and a Plano lens on the right as a control. After 12 h (7 chickens, lens diffuser placed from 10 PM to 10 AM) and 7 days (11 chickens) of lens wear, the chicks were sacrificed with an overdose of carbon dioxide. Eyes were removed and were hemisected near the equator in the equatorial plane. The retinas were dissected out.

### Refraction measurements in chicken model

Refractive errors and ocular biometric dimensions were determined by retinoscopy and A-scan ultrasonography using a 25 MHz transducer (V324-SU; Panametrics) and sampled at 250 Mhz with Agilent 1067A Acqiris DP110 Digitize (Resolution up to 50 µm) respectively. Streak retinoscopy (Heine, Gilching, Germany; Beta 200 Streak Retinoscope Set 2.5 V) was used to measure refractive error. Refractive errors and biometric measurements were conducted both before and after minus lens wear. Retinoscopy was used to measure the refractive status.

## Immunocytochemistry

### Antibodies

Rabbit anti-phospho-Ser276 (Ser276-P, 1:1000, kindly provided by Dr. John O’Brien, The University of Texas) was used for retinal labeling (Kothmann et al., 2007). This antibody has been previously tested in perch (Kothmann et al., 2007), rabbit (Kothmann et al., 2009; Kothmann et al., 2012), and mouse retinas (Ivanova et al., 2015). Rabbit polyclonal antibody anti-Cx36 (1:1000; Invitrogen, Carlsbad, CA; catalog no. 36-4600, RRID:AB_2533260) was used in previous studies of the retina in mammals (Ivanova et al., 2015) and chicken (Kihara et al., 2009). The specificity of the polyclonal antibody anti-Cx36 was verified by Western blot in chicken retinas. However, in this experiment, mouse anti-Cx35/36 (1:1000, EMD Millipore, Burlington, MA; Cat# MAB3045, RRID:AB_94632) was used for chicken retina labeling. The mouse anti-Cx35/36 antibody is co-localized with the labeling of rabbit anti-Cx36 antibody in all layers of the IPL (Supplemental Fig. S1). In the IPL, 98.4 ± 0.02% mouse anti-Cx35/36 puncta were co-localized and associated with rabbit anti-Cx36 labeled plaques in the chicken retina (5 retinas × 3 samples = 3584 detectable plaques). Rabbit polyclonal antibody anti-Prox1 (1:1000; BioLegend; San Diego, CA, catalog no. USA PRB-238C; RRID:AB_2801255) was used in previous studies of AII ACs in murine (Perez de Sevilla Muller et al., 2017) and human retinas (Hoshino et al., 2017).

For consistency, all chicken retinas were taken from the pecten’s dorsal side (only the middle section) around 10 AM under illuminance (500–700 Lux). The retinal pieces, attached to filter paper (RGCs up) were submersion-fixed in 2% N-(3-dimethylaminopropyl)-N′-ethylcarbodiimide hydrochloride (“carbodiimide” or “EDAC”; E7750, Sigma-Aldrich, St. Louis, MO in 0.1 M phosphate buffer (PB), pH 7.5 for 30 min at room temperature. After fixation, the retinas were separated from the filter paper and washed with phosphate buffered saline (PBS) before being washed extensively with a 0.1 M phosphate buffer (PB, pH 7.4) and blocked with 3% donkey serum in 0.1 M PB 0.5% Triton-X 100 and 0.1% NaN_3_ overnight. The antibodies were diluted in 0.1 M PB with 0.5% Triton-X 100 and 0.1% NaN3, containing 1% donkey serum. The tissues were incubated in primary antibodies for 3–7 days at 4°C and, after extensive washing, incubated in secondary antibodies overnight at 4°C (Pan & Massey, 2007). The secondary antibodies used were donkey anti-mouse Cy-3 (1:200) and donkey anti-rabbit 488 (Jackson Immunoresearch Laboratories, West Grove, PA; 1:200). After washing with 0.1 m PB, the tissues were mounted in Vectashield (Vector Laboratories, Burlingame, CA) for observation.

### Western blotting

Chicken retinal proteins were resolved on sodium dodecyl sulfate polyacrylamide gel electrophoresis and then transferred to polyvinylidene fluoride membranes by standard procedures. After blocking with Tris-buffered saline containing 0.1% Tween-20 and 5% skim milk powder for 1 h at 37°C, the membranes were incubated with primary antibody overnight at 4°C. Mouse anti-glyceraldehyde-3-phosphate dehydrogenase (anti-GAPDH, 1:5000, Sigma Millipore, Burlington, MA, Cat# CB1001, RRID:AB_2107426) antibody was used as a loading control.

### Retinal slice preparation and injection of neurobiotin

The retina was carefully separated from the sclera in a chamber with extracellular solution, containing (in mM): 120 NaCl, 2.5 KCl, 25 NaHCO_3_, 0.8 Na_2_HPO_4_, 0.1 NaH_2_PO_4_, 1 MgCl_2_,2 CaCl_2_, and 5 d-glucose. The bath solution was continuously bubbled with 95% O_2_–5% CO_2_ at 32°C. A piece of central retina was embedded in type VII agarose (Sigma) and cut with a vibratome (Leica VT1200S) to 200 *μ*m thickness. Retinal slices were transferred to the recording chamber and superfused with the oxygenated extracellular solution (flow rate 1–2 ml/min) for neurobiotin injection.

The AII-like ACs identified were similar in their morphology to those of the mouse retina, having thick lobular appendages in the OFF sublamina of the IPL and descending arboreal processes to the ON sublamina of the IPL (Lee et al., 2015). The AII-like ACs were visualized at 40× magnification and were impaled under visual control, using pipette tips filled with 4% neurobiotin (Vector Laboratories, Burlingame, CA) and 0.5% Lucifer Yellow-CH (Molecular Probes, Eugene, OR) in double-distilled water and then backfilled with 3 M KCl. The electrode resistance was ∼100 MΩ. The impaled cells were then injected with a biphasic current (+1.0 nA, 3 Hz). After the injection, the retinal pieces were fixed in 4% paraformaldehyde for at least 5 min. The tissues were incubated overnight at 4°C in 0.1 M phosphate buffer with 0.5% Triton-X 100 and 0.1% NaN_3_ containing 1% donkey serum and then, after extensive washing, incubated in Cy3-conjugated streptavidin (Invitrogen, Carlsbad, CA) 1:200 overnight at 4°C. The tissues were mounted in Vectashield (Vector Laboratories) for observation.

### Imaging and data quantification

Images of whole retinal mounts were acquired on a ZEISS LSM 800 with an Airyscan (Zeiss, Thornwood, NY) confocal microscope using a 63× objective (N.A. 1.4). The instrument’s optical resolution was 120 nm in the *x*- and *y*-axes, and 350 nm in the *z*-axis and all channels (488 and Cy3) were superimposed. *Z*-axis steps were usually 0.35 *μ*m. Based on the optical resolution of 120 nm in the *x*- and *y*-axes. Four fields from each retina (the dorsal side of the pecten) were analyzed. The retinas were imaged under identical acquisition conditions, including laser intensity, pinhole, photomultiplier amplification, and *z*-stack step size. The detector gains and offset parameters were optimized. All the settings remained unchanged after optimization for each experiment to avoid imaging biases. The region of interest (ROI) identification was accomplished by using Image J software (Image J, 1.52i, RRID:nif-0000-30467). ROI borders were defined by a 20% intensity threshold in the channel and a size threshold of 0.01 *μ*m^2^ as well. The analysis was conducted as described previously (Kothmann et al., 2009; Kothmann et al., 2012; Ivanova et al., 2015). The data were presented as mean ± s.e.m. unless otherwise indicated. The ratio of the mean density of Ser276-P to Cx35 immunofluorescence was calculated for each of ROIs and averaged across all ROIs in all images per condition. In this way, it was possible to collapse the phosphorylation data into one value per condition per animal to perform statistical analysis.

Statistical analyses were performed using Origin software (OriginLab, Northampton, MA) and SPSS version 25 (Armonk, NY). Statistical significance (*P* < 0.05) was determined by using Wilcoxon Signed Rank test.

## Results

### Seven days LIM and measurement of refractive errors in chicken eye

**Fig. 1.**
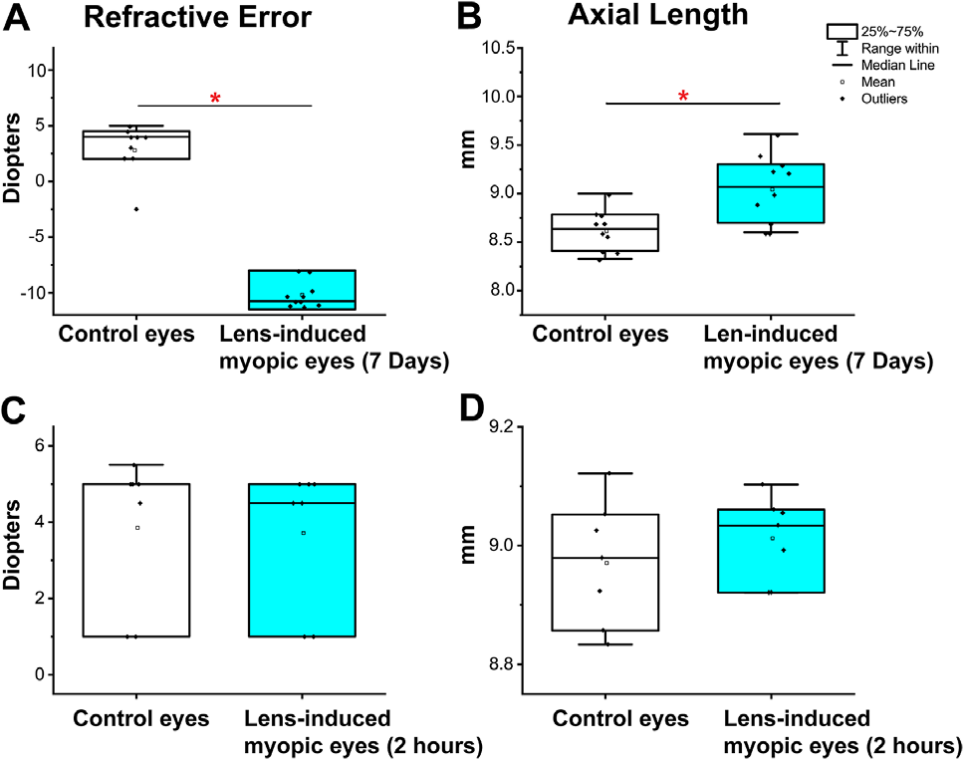
Measurement of Lens-induced refractive errors in chicken eyes by streak retinoscopy and A-scan ultrasonography. (A) Refractive errors of chicken eyes were measured by streak retinoscopy. LIM method was employed for 7 days to produce precise and consistent myopia in chicken eyes (−10.08 ± 0.89 D, mean ± s.e.m., *P* < 0.05, *n* = 10). (B) AL of the chicken eye was measured from the cornea’s surface to the RPE layer by A-scan ultrasonography. The AL of LIM eyes was 9.06 ± 0.12 mm, in contrast to 8.63 ± 0.08 mm (mean ± s.e.m., *P* < 0.05, *n* = 10), of the contralateral control eyes, an average increase in AL of 0.29 ± 0.12 mm. (C) 12 h LIM did not affect refractive errors of chicken eyes by streak retinoscopy (control, 3.86 ± 0.75 D; 12 h LIM, 3.71 ± 0.71 D, *P* = 0.36, *n* = 7). (D) AL of the chicken eye had no significant change after 12 h LIM. The AL of control eyes was 8.97 ± 0.04 mm, in contrast to the AL of 12 h LIM (9.01 ± 0.03 mm, *P* = 0.06, *n* = 7).

Retinoscopy revealed that the refractive errors after 7 days LIM and control chicken were −10.1 ± 0.9 D and +2.8 ± 0.9 D (mean ± s.e.m., *P* < 0.05, *n* = 10), respectively (Fig. 1A).

AL measurements from the cornea’s surface to the RPE layer supported the findings of underlying changes in the chicken eyes’ refractive state. The AL of the LIM eyes was 9.06 ± 0.12 mm, in contrast to 8.63 ± 0.08 mm (*P* < 0.05, *n* = 10) (Fig. 1B) of the contra-lateral non-LIM control eyes, which represented an average increase in AL of 0.29 ± 0.12 mm in the LIM eyes. Eyes with refractive errors from −8 to −12 D, confirmed by retinoscopy measurement, were selected for characterization of changes in phosphorylation of Cx35 in the retina.

As to control, 12 h short-term LIM had no significant effects on both refractive errors (control eyes: 3.86 ± 0.75 D; 12 h LIM eyes, 3.71 ± 0.71 D, *P* = 0.36, *n* = 7) (Fig. 1C) and AL (control eyes: 8.97 ± 0.04 mm, 12 h LIM: 9.01 ± 0.03 mm, *P* = 0.06, *n* = 7) (Fig. 1D).

### Specificity of anti-Cx35 phosphorylation antibodies in chicken retinas

**Fig. 2.**
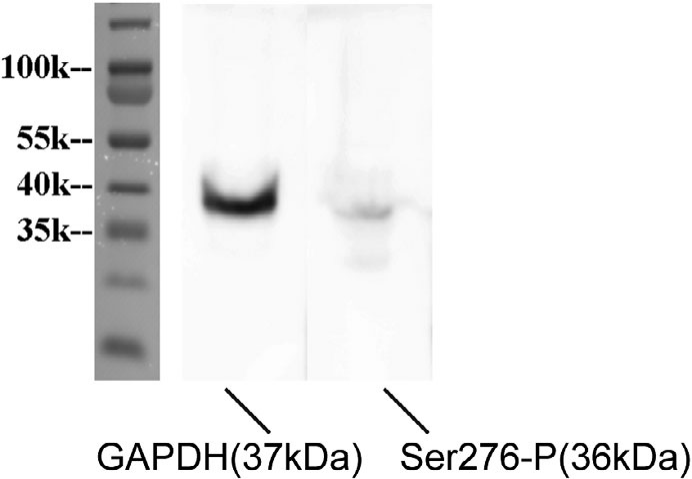
Specificity of the phospho-Cx35/36 antibodies. Western blot analysis of anti-phospho-Ser276 (Ser276-P) of Cx35/36 antibody in the chicken retina (36 kDa). Anti-GAPDH antibody (1:5000, 37 kDa) was used as the loading control.

As phosphorylation of Cx35 at Ser276 is involved in the regulation of coupling in retinal neurons, Ser276-P antibodies were used to visualize phosphorylated Cx35 and, thereby, identify coupled gap junctions. The specificity of Ser276-P antibodies was determined with Western blots against phosphorylated Cx35 proteins in the chicken retina (Fig. 2 and Supplement Fig. S2). Probing of the blot with polyclonal anti-phospho-Ser276 antibody revealed a robust and specific reaction at approximately 36 kDa. GAPDH was loaded as a positive control at 37KDa.

The complete blot is shown in the Supplemental Fig. S2.

**Fig. 3.**
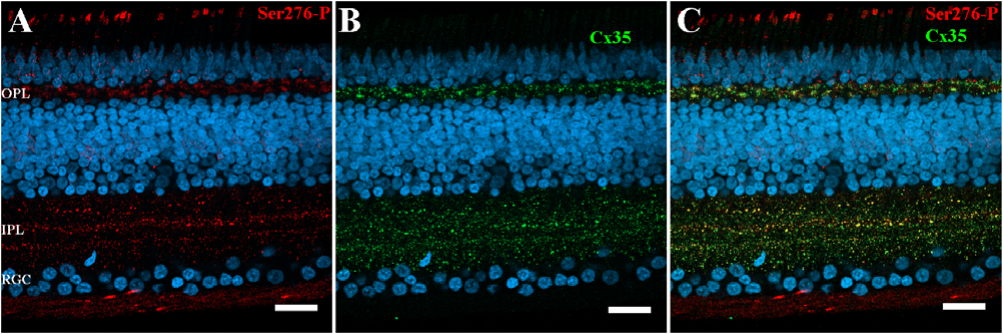
Ser276-P antibody labeling pattern in the chicken retina after 7 days −10D defocus. (A–C) Labeling pattern of anti-phospho-Ser276 antibody in the 7 days LIM chicken retina. (A) Ser276-P antibody labeled(red) abundant punctate structures in both IPL and OPL. The labeling is also observed in the optic fiber layer, in the retinal pigment epithelium layer, and photoreceptor inner/outer segments. (B) Labeling with polyclonal Cx35 antibody (green) shows labeling of Cx35 also in the OPL and IPL. (C) The merged image of A and B shows multiple plaques of Ser276-P co-localized with Cx35 antibody labeling (yellow). Scale bar is 20 *μ*m.

In the IPL, 93.4 ± 0.02% Ser276-P positive puncta were co-localized with Cx35-labeled plaques in the chicken retina (5 retinas × 3 samples = 13,258 detectable in Ser276 phosphorylated plaques), demonstrating that the antibody was highly specific for Cx35 in the chicken retina (Fig. 3A–3C). There are also prominent Cx35-labeled structures in the OPL, and it was observed that the smaller ones were most frequently labeled for Cx35. Ser276-P positive puncta were also observed in the optic fiber layer, retinal pigment epithelium layer, and photoreceptor inner/outer segments.

### AII-like ACs in the chicken retina

Although AII ACs are well characterized in the mammalian retina (MacNeil & Masland, 1998), this is not the case in the chicken retina.

**Fig. 4.**
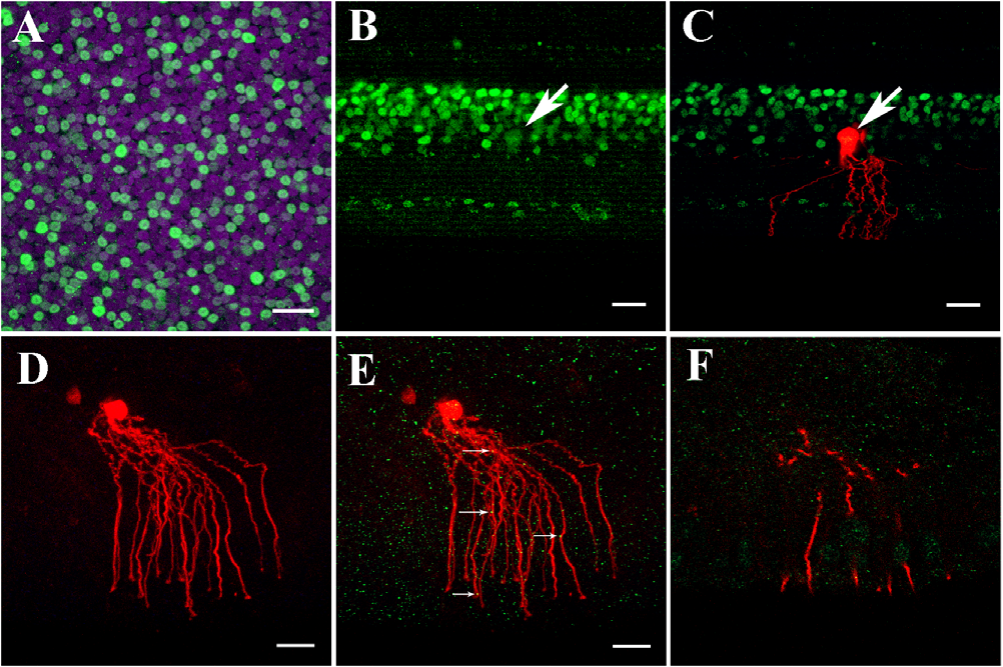
AII-like ACs in chicken retina expressed Cx35. (A) Prox1 immunostaining in the chicken retina. Prox1-immunoreactive AII-like AC bodies and bipolar cells in the INL in a whole-mounted chicken retina. (B) Prox1 immunoreactivity in AII AC and bipolar cell bodies in INL in the vertical section. Prox1-immunoreactive AC bodies are present at the bottom of the INL. (C) Prox1-immunoreactive AII-like AC’s cell body co-localized with neurobiotin injection. The AII-like AC was similar to AII AC’s morphology in the mouse retina, having thick lobular appendages in the OFF sublamina of the IPL and descending arboreal processes to the ON sublamina of the IPL. (D) Cy3-labeled AII-like AC in the chicken retina (200 *μ*m slice) was targeted and injected with neurobiotin in the INL to show single AII-like AC’s soma–dendritic morphology in *Z*-stack by three-dimensional reconstruction image. The cell had a round cell body with distinct dendritic trees. The proximal dendrites near soma had lobular appendages. The distal processes overlapped along central-to-peripheral axes. In addition, the injected cell is coupled with the nearby cells. (E) AII-like AC double-labeled with anti-Cx35 (green) puncta located predominately on the arboreal dendrites (as indicated by white arrows). (F) Single plane of the confocal image showing Cx35 puncta on segments of AII like AC dendrites. A–C: Scale bar is 20 *μ*m; D–F: Scale bar is 5 *μ*m.

The transcription factor Prox1 polyclonal antibody was applied to label AII-like ACs in the chicken retinas. Prox1-immunoreactive cell bodies were observed in the proximal inner nuclei layer (INL), the middle and distal INL and the ganglion cell layer in the whole mount-retinas (*n* = 3) (Fig. 4A). Prox1-immunoreactive cell bodies with large round soma, suggesting that they were ACs, were located in the bottom of the INL (Fig. 4B). Following injection with neurobiotin, the cells exhibited homologous coupling to other AII-like ACs (*n* = 5) (Fig. 4C and 4D), displaying round cell bodies with distinct dendritic trees. The proximal dendrites near the soma had lobular appendages. The distal processes overlapped along the central-to-peripheral axes. The Prox1-immunoreactive AII-like AC cell bodies co-localized with neurobiotin injected ACs and displayed similar morphology to the AII ACs in the mouse retina, having thick lobular appendages in the OFF sublamina of the IPL and descending arboreal processes to the ON sublamina of the IPL. Thus, the AII-like ACs can be identified by injection method.

### Cx35 localization in the chicken retina

**Fig. 5.**
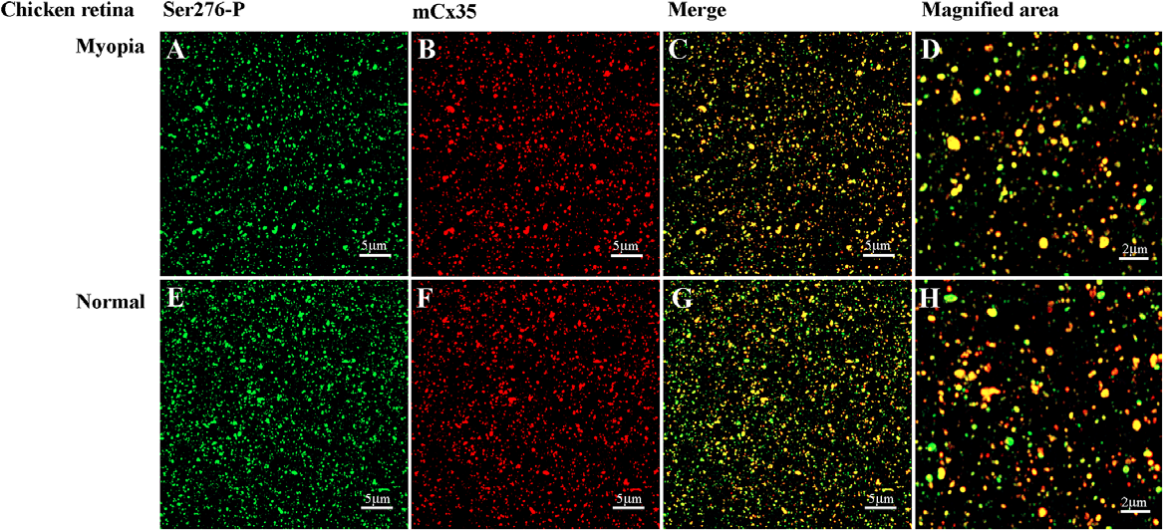
Phospho-Ser276 antibody recognizes Cx35 in the whole mount chicken retina of 7 days LIM model. (A–D [−10D treated]) Confocal stack sections in stratum 5 of the IPL in a myopic chicken retina after 7 days LIM treatment: Cx35, labeled in red, and its phosphorylated form, Ser276-P (green), are present with similar punctate labeling. The magnified areas showed the merged images of phosphorylated Ser-276 and Cx35, reflected by yellow color. (E–H [non-LIM]) Ser276-P antibody recognizes Cx35 in the non-LIM control chicken retina. Images are 2 *μ*m deep stacks.

Most Cx35 puncta (98.7 ± 0.04%) were located in the IPL of the chicken retina. Ser-276 (96.7 ± 0.03%) puncta were also predominantly located in the same layer (Fig. 5). Cx35 also co-localized on the AII-like ACs (*n* = 5) (Fig. 4E and 4F). Therefore, the follow-up experiments focused on the phosphorylation state of Cx35 in the ON sublamina layer in the IPL, and only the S5 layer close to RGC was observed in the study.

### Cx35 phosphorylation in the chicken retina of 7 days LIM model

**Fig. 6.**
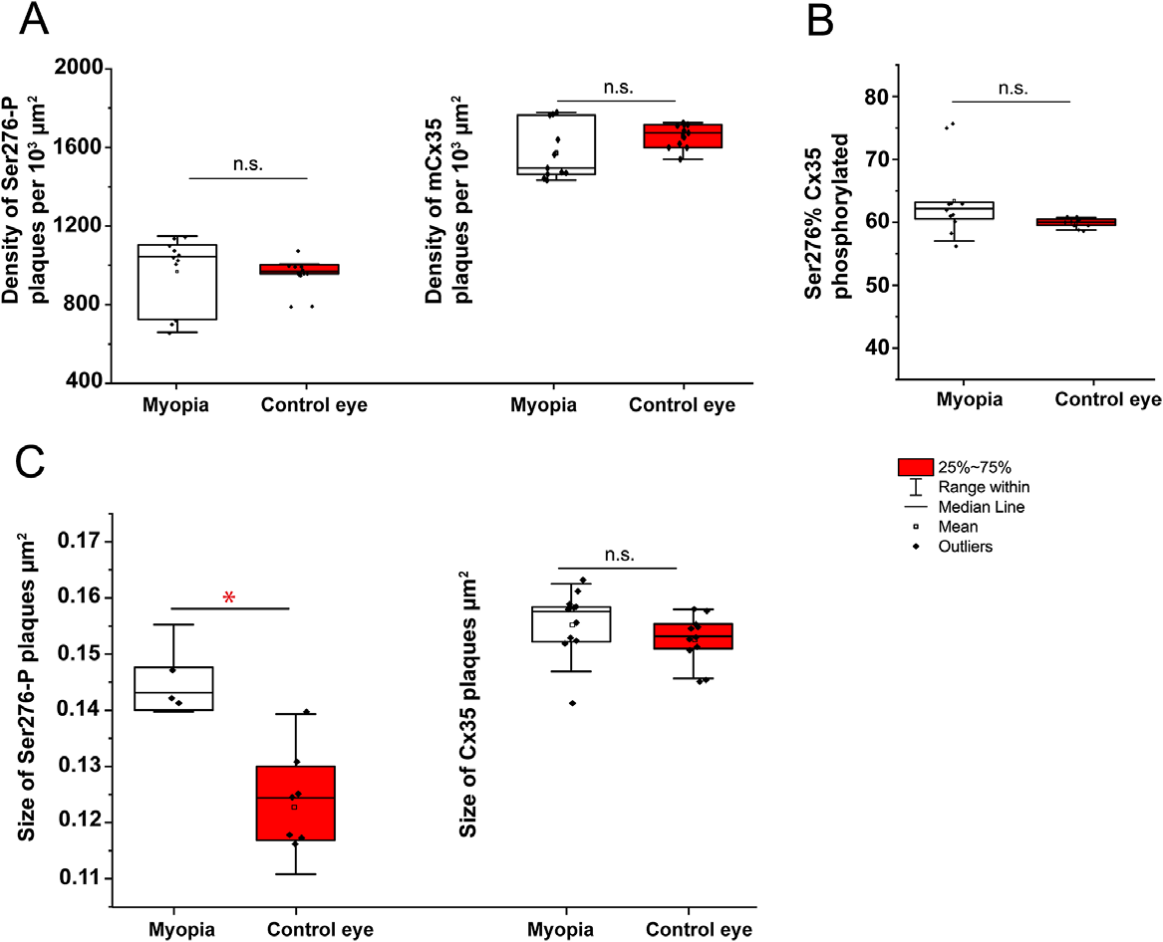
Quantification of phosphorylation Ser276 of Cx35 gap junctions in 7 days lens-induced chicken myopic retinas. The size of Ser276-P plaques (C) was significantly increased in the myopic retina compared to that of control retinas in 7 days LIM-treated eyes. The density (plaques number per 10^3^ *μ*m^2^) of phosphorylation reflected by detectable Ser276-P labeling (A), the density (plaques number per 10^3^ *μ*m^2^) of Cx35 labeling (A), the percentage of Ser276-P of Cx35 phosphorylated rate (B), and the size of Cx35 plaque (C) did not differ between retinas of LIM and non-LIM eyes. The data are presented as averages; error bars are s.e.m.. Significance is based on Wilcoxon Signed Ranks Test, where * indicates 0.01 < *P* < 0.05 and n.s. indicates *P* > 0.05.

The eye growth had fully compensated for −10D defocus after 7 days, the other eye as control with the Plano lens. Ser276-P antibody recognized the Ser276-phosphorylated form of Cx35 in S5 of the IPL of the chicken retina was observed after 7 days LIM. There was no significant difference in density (number of immunoreactive plaques per 10^3^ *μ*m^2^) between LIM myopic and non-LIM control retina (Fig. 6). Following evaluation of 8325 individual plaques in the LIM myopic eyes (*n* = 11) and 7884 plaques in non-LIM control eyes (*n* = 11), a significant difference in the area of S276 plaques was observed (non-LIM control retina 0.122 ± 0.002 *μ*m^2^; LIM retina 0.144 ± 0.001 *μ*m^2^; *P* = 0.011).

The density of Cx35-positive puncta per 10^3^ *μ*m^2^ in the non-LIM control retinas also did not differ significantly from the LIM myopic retinas. There was also no significant difference in the percentage of Ser-276 of Cx36 phosphorylated between LIM myopic and non-LIM control retinas. Following evaluation of individual plaques in retinas of LIM myopic eyes (*n* = 11) and plaques in non-LIM control eyes (*n* = 11), no difference in the area covered per punctum of Cx35 across groups was observed (Table 1).

**Table 1.** Connexin35 phosphorylation in the chicken retina of 12 hrs and 7 days’ LIM model

Treatment	Ser276 plaque number examined	Density of Ser-276 immuno-reactive plaques (per 10^3^ *μ*m^2^)	*P*	Ser276 plaque area (*μ*m^2^)	*P*	Density of Cx35-positive puncta (per 10^3^ *μ*m^2^)	*P*	Percentage of Ser-276 of Cx35 phosphorylated (%)	*P*	Cx35 plaque number examined	Area covered per punctum of Cx35 (*μ*m^2^)	*P*
12 h LIM (*n* = 7)	5680	1183 ± 41	0.02	0.141 ± 0.003	0.01	1693 ± 27	0.42	70.3 ± 0.6	0.01	6132	0.156 ± 0.002	0.36
12 h control (*n* = 7)	6013	1015 ± 22		0.124 ± 0.002		1686 ± 21		58.9 ± 1.2		7435	0.155 ± 0.002	
7d LIM (*n* = 11)	8325	940 ± 41	0.8	0.144 ± 0.001	0.01	1663 ± 49	0.12	64.5 ± 8.9	0.1	10505	0.156 ± 0.002	0.34
7 day control (*n* = 11)	7884	964 ± 88		0.122 ± 0.002		1578 ± 42		58.5 ± 1.1		8697	0.154 ± 0.002	

Summary of the data and statistics difference of Cx35 phosphorylation in the chicken retina of 12 h and 7 days LIM model.

All the data showed in mean ± s.e.m.

### Cx35 phosphorylation in the 12 h LIM chicken retina

**Fig. 7.**
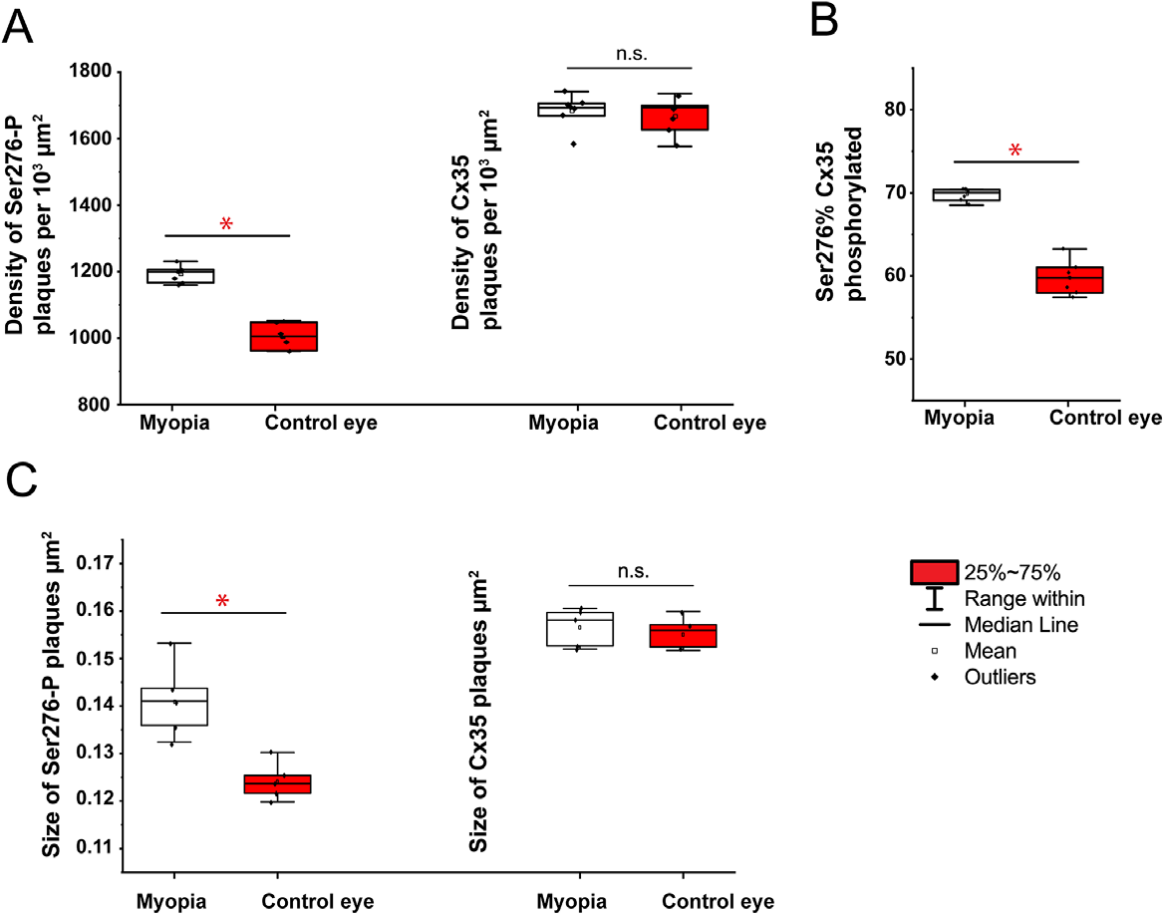
Quantification of phosphorylation of Cx35 gap junctions in 12 h lens-induced chicken myopic retinas. In the 12 h LIM chicken eye, the density (plaques number per 10^3^ *μ*m^2^) of phosphorylation reflected by detectable Ser276-P labeling (A), the percentage of Ser276-P of Cx35 phosphorylated rate (B), and the size of Ser276-P plaque (C) were significantly increased in the LIM retina compared to non-LIM control retinas. The density of Cx35 labeling (A) and the size of Cx35 plaque (C) had no significant difference between the LIM retinas compared to non-LIM control retinas. The data are presented as averages; error bars are s.e.m. Significance is based on Wilcoxon Signed Ranks Test, where * indicates 0.01 < *P* < 0.05 and n.s. indicates *P* > 0.05.

After 12 h LIM, the defocus induced changes in chicken retinal activity are in full flow. Thus, Cx35 phosphorylation had a significant difference in density of Ser-276 puncta between LIM myopic (1183 ± 41 per 10^3^ *μ*m^2^, *n* = 7) and control retina was observed (1015 ± 22 per 10^3^ *μ*m^2^, *n* = 7, *P* = 0.02) (Fig. 7). Following evaluation of puncta in retinas of LIM myopic eyes and control retinas), the area covered per punctum was greater in LIM retinas (0.141 ± 0.003 *μ*m^2^, *n* = 7) than controls (0.124 ± 0.002 *μ*m^2^, *n* = 7, *P* = 0.02) (Table 1).

However, although the density per of mCx35-positive puncta per 10^3^ *μ*m^2^ in the control retina did not differ significantly from the LIM retina, the percentage of Ser-276 of Cx35 phosphorylated was higher in LIM myopic retinas than non-LIM controls. Following evaluation of plaques in retinas of LIM and control eyes, no significant difference in the area covered per Cx35 plaque across groups was observed (Table 1 and Fig. 7).

## Discussion

In our previous study in the myopic mouse retina, phosphorylation Ser-293 of Cx36 gap junctions in AII ACs increased, while the expression of Cx36 remained unchanged. The increased phosphorylation of Cx36-Ser276 (293 in mammals) implies that gap-junctional coupling is extended. Therefore, the increase in phosphorylation of Ser293 in AII-ACs in the myopic mouse retina indicates that extended coupling of AII ACs involved in the myopia development of mammals. The AII AC is an interneuron cell in the scotopic (night-vision) pathway in the retina and also contributes to photopic (daylight vision) in cone-dominated primate retinas (Sohl et al., 2005). However, considering that an equivalent to AII AC has not been identified in the retinas of non-mammals, including chicken, the cells responsible for retinal modulation of axial elongation in the chick eye remain to be identified.

The lens-induced chicken myopia model provides an excellent means to study mammals and humans’ refractive development. The main finding of this study is that the size of Ser-276- phosphorylated plaques of Cx35 in the sublamina 5 of the IPL increased in 7 days LIM chicken retinas (The eye growth had fully compensated for 7 days’ -10D lens. The refractive errors of the eyes were approximate -10D). The amount of phosphorylation of Ser276 and the size of Ser276 phosphorylated Cx35 plaques were significantly increased after 12 h in LIM chicken myopic retinas (when the defocus-induced changes in chicken retinal activity were fully activated (Swiatczak et al., 2019). It is recognized that the retina can sense the focus of images (Pan, 2019), and then generate signals to regulate eye growth (Smith et al., 2005). LIM in chickens is reported to affect retinal activity, occurring around several hours to 1–2 days after the intervention (Wildsoet & Wallman, 1995).

### The rationale for the chick myopia model

In myopia research, several findings related to neural control of eye growth were first observed in the chick model and subsequently applied to other mammalian or human eyes (Norton, 1999). Diurnal fluctuations in eye size, which are mechanistically significant for myopia, initially observed in chicks (Weiss & Schaeffel, 1993), were later reported in marmosets (Nickla et al., 2002) and humans (Stone et al., 2004). Chicks have precise, visually guided emmetropization (Wallman et al., 1978; Schaeffel et al., 1988; Shi et al., 2020). Compared to other laboratory animals, the chick has superior optical quality and faster and stronger refractive responses than those of the mouse. Within 1–2 weeks, myopia can develop sufficiently to replicate myopia models for data collection (Schaeffel & Feldkaemper, 2015). Compared to other mammalian eyes, the chick eye has disadvantages, including scleral ossicles and cartilage, striated intraocular muscles, and a pecten projecting freely from the retina into the vitreous body. Yet, it still offers many advantages for myopia research. Most importantly, the fundamental neural systems linking vision to eye growth in chicks and mammals share many common features (Schaeffel & Feldkaemper, 2015).

However, the retinal circuitry of chicken is different from that of mammals. For example, the rod pathways via the unique rod bipolar to AII ACs and then to the cone bipolar cell network appear to be absent in the chicken retina (Seifert et al., 2020). This is probably because AII ACs in mammalian retinas are highly specialized for the rod-driven pathways through the retina. This is in comparison to nonmammalian species so far investigated, including fish (Scholes, 1975; Stell et al., 1975; Ishida et al., 1980), salamander (Lasansky, 1973), and turtle (Dacheux, 1982; Kolb, 1982), which appear to have mixed rod-cone bipolar cells and no purely rod bipolar cells. Besides, the structures of photoreceptors (Baylor & Hodgkin, 1973; Kram et al., 2010) and horizontal cells (Burkhardt, 1995; Tanabe et al., 2006) in turtles and birds are very similar. Thus, the rod-AII pathway in the chicken retina may also differ from that of mammals.

In this study, bistratified, narrow-field AII-like ACs were easily identified by their distinctive dendrite morphology and soma location via neurobiotin injection. The Prox1-immunoreactive labeling strongly indicated that the cells injected were AII-like ACs. However, to confirm these cells are AII ACs in chicken retinas, further work such as GABA or Glycine immunoreactive labeling will be required. The anatomical connections between the AII-like ACs and other rod and/or cone bipolar cells also need to be investigated.

### Gap junction and phosphorylation of Cx35 in ACs

Intercellular signal communication and modulation of visual signals in the retina can be achieved via gap junctions (Bloomfield & Volgyi, 2009). Gap junctions are highly plastic to tune retinal microcircuits and process visual information under different conditions (O’Brien & Bloomfield, 2018). Cx35/36 is the predominant subunit of gap junctions in the retina (Bloomfield & Volgyi, 2004). Myopia induction increased Ser293 in the rod-dominant mouse retina (Density of Ser293 and size; Banerjee et al., 2020). The mouse retina may need more functional Cx36 to filter noise under myopia status because of the rod-dominant retina. The area of Ser-276 in the chick is only half that previously observed in the mouse retina. These differences may relate to the plasticity of gap junctions in different species allowing them to play varied roles in the visual signaling process.

In the retina of LIM eyes in chickens, the size of Ser276-P plaques was significantly increased in S5 of IPL after both 12 h and 7 days of −10D defocus. The amount of phosphorylation of Ser276-P also significantly increased after 12 h with the increased size of Ser276-P plaques. The results showed that in the early stage when the defocus-induced by lens changes in retinal activity were in full flow, an increase of phosphorylation of Ser276-P in Cx35 was induced. After lens-compensation for 7 days, the amount of phosphorylation of Ser276-P returned to normal, but the plaques’ size remained greater than those of control eyes. Preliminary experiments revealed that the size of Ser276-P plaques decreased in hyperopia (unpublished data) compared with control mouse retinas. This may suggest that phosphorylation of Cx35/36 was involved with the coding of differential signaling of myopic/hyperopic defocus. We further hypothesize that myopia development started in the retina where AII-like ACs might act as a midway to deliver the signaling of defocus from RGCs to the sclera.

In conclusion, this study showed an increase in phosphorylation of Cx35 in the cone-dominant retina of the LIM myopic chicken. Further studies are needed to clarify the complex etiology and nature of physiological remodeling in myopia development.

### Limitations of the study

Chicken does not employ the same retinal circuitry as mammals, which have a specialized rod pathway via the unique rod bipolar AII AC-cone bipolar cell network. AII, A8, and A17 ACs may only exist in mammalian species. Even Prox1-immunoreactive AII-like ACs were identified and indicated the possible AII ACs in the chicken retina. AII-like ACs were still named in the research. Because of the difference of retinal circuit, findings from the chicken cannot be matched with that of the mouse. The gap junction state (Cx36) in the research was estimated from the phosphorylation of Cx36. However, this is an indirect estimation, and future studies involving gap junction permeable tracer coupling and electrophysiological recording need to be done.

Therefore, the findings from the chicken myopia model should be cautiously applied to human myopia research.

## Supplementary material

For supplementary material accompanying this paper visit http://dx.doi.org/10.1017/S0952523821000079. click here to view supplementary material
