# Functional connexin35 increased in the myopic chicken retina—CORRIGENDUM

**DOI:** 10.1017/S0952523821000109

**Published:** 2021-07-23

**Authors:** Seema Banerjee, Qin Wang, George Tang, ChungHim So, Sze Wan Shan, King Kit Li, Chi-Wai Do, Feng Pan

**Keywords:** myopia, gap junction, retina, amacrine cell

In the original version of Banerjee et al., 2021, author Qin Wang’s name was misspelled.

The original article has been updated.
