# Supplementary figures and images for: Functional connexin35 increased in the myopic chicken retina
Source: Vis Neurosci. 2021 May 14;38:E008. doi: 10.1017/S0952523821000079 (PMC8167454; doi:10.1017/S0952523821000079)

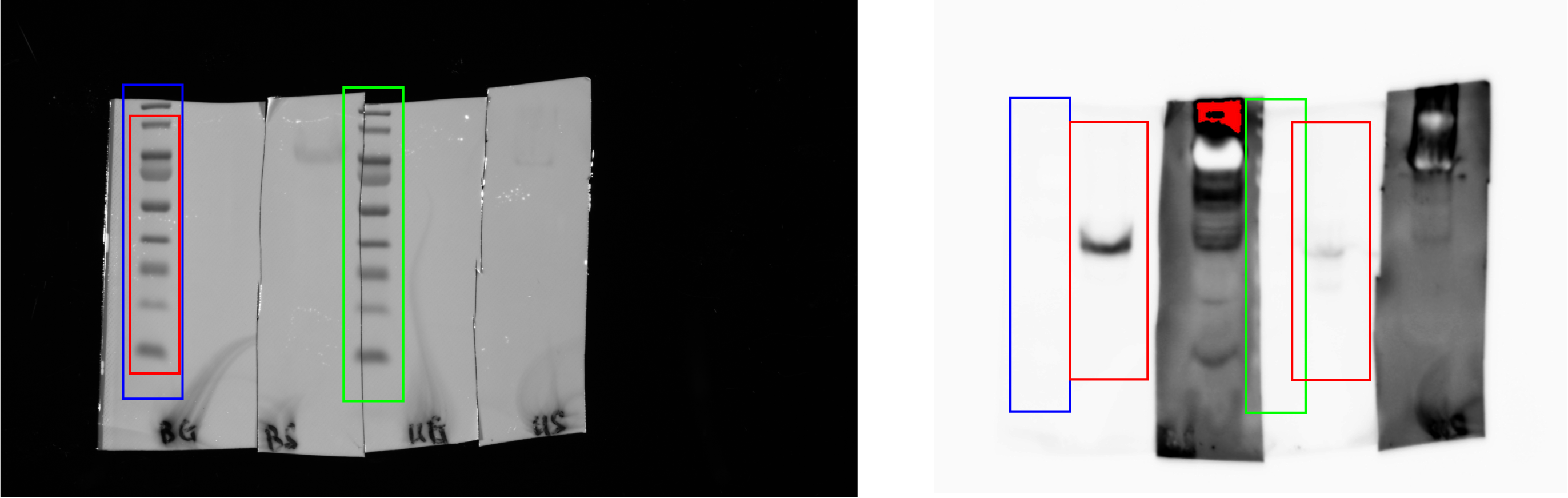

Supplement: Supplementary file 1 [file vnssup.zip › S0952523821000079sup001.tif]

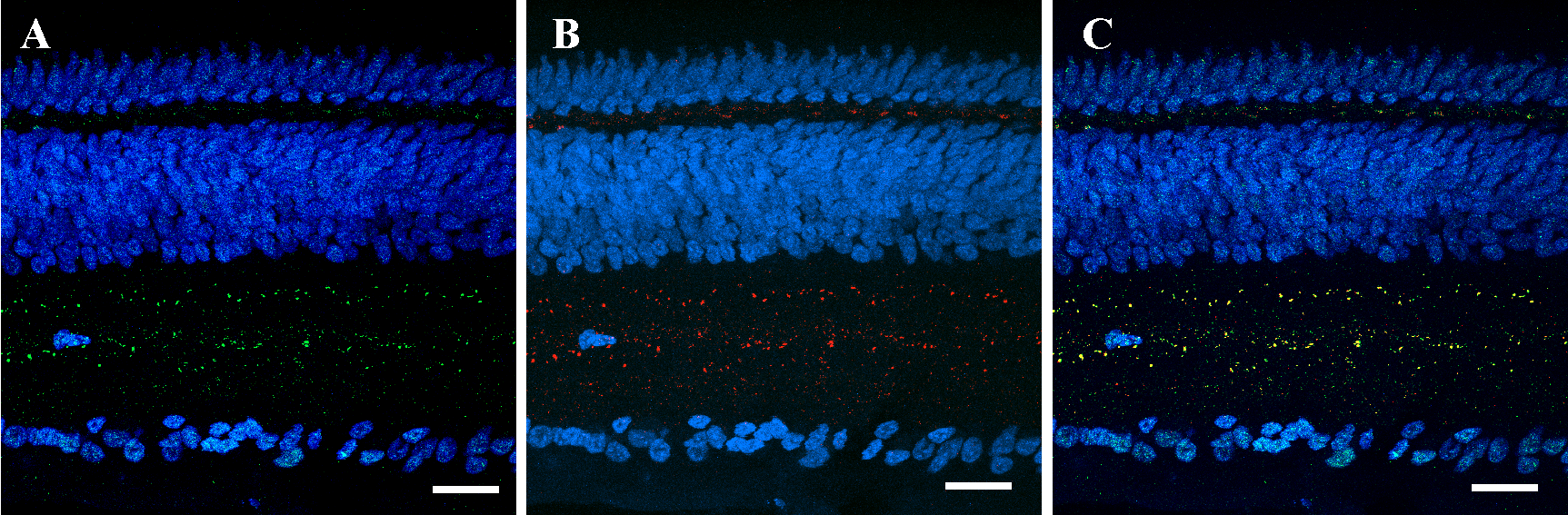

Supplement: Supplementary file 1 [file vnssup.zip › S0952523821000079sup002.tif]
